# Alginic Acid Polymer-Hydroxyapatite Composites for Bone Tissue Engineering

**DOI:** 10.3390/polym13183070

**Published:** 2021-09-11

**Authors:** Rebecca Sikkema, Blanca Keohan, Igor Zhitomirsky

**Affiliations:** Department of Materials Science and Engineering, McMaster University, Hamilton, ON L8S4L7, Canada; sikkemar@mcmaster.ca (R.S.); keohanb@mcmaster.ca (B.K.)

**Keywords:** alginate, hydroxyapatite, composite, gel, film, coating, scaffold, biocement, nanoparticle

## Abstract

Natural bone is a composite organic-inorganic material, containing hydroxyapatite (HAP) as an inorganic phase. In this review, applications of natural alginic acid (ALGH) polymer for the fabrication of composites containing HAP are described. ALGH is used as a biocompatible structure directing, capping and dispersing agent for the synthesis of HAP. Many advanced techniques for the fabrication of ALGH-HAP composites are attributed to the ability of ALGH to promote biomineralization. Gel-forming and film-forming properties of ALGH are key factors for the development of colloidal manufacturing techniques. Electrochemical fabrication techniques are based on strong ALGH adsorption on HAP, pH-dependent charge and solubility of ALGH. Functional properties of advanced composite ALGH-HAP films and coatings, scaffolds, biocements, gels and beads are described. The composites are loaded with other functional materials, such as antimicrobial agents, drugs, proteins and enzymes. Moreover, the composites provided a platform for their loading with cells for the fabrication of composites with enhanced properties for various biomedical applications. This review summarizes manufacturing strategies, mechanisms and outlines future trends in the development of functional biocomposites.

## 1. Introduction

Alginic acid (ALGH) is a natural biocompatible and biodegradable polymer, which is widely used for various biomedical applications. Low-cost natural alginates can be derived from brown marine algae. In physiological conditions, ALGH takes the form of a highly soluble sodium salt (ALGNa). ALGH is a polysaccharide, containing mannuronate and guluronate monomers [1]. The anionic properties of ALGH in solutions are attributed to COO^−^ groups of the individual monomers. At low pH, the precipitation of ALGH gel is observed. The gel-forming properties of ALGH provide a versatile platform for the development of advanced drug delivery technologies. ALGH can be crosslinked with metal ions or organic molecules for the fabrication of gels with enhanced mechanical properties, chemical stability, improved cell compatibility, surface wettability and desired morphology [2,3].

ALGH is widely used for the fabrication of biomedical scaffolds for bone tissue engineering [4], capsules, microspheres and stents for drug delivery [5,6,7] and adsorptive beads for protein separation and purification [8]. Innovative studies during recent years have led to various applications of ALGH fibers for wound dressing, drug delivery, tissue engineering and biosensors [9,10]. This biopolymer is a promising material for surface modification of biomaterials. ALGH has been used for the development of protein resistant coatings [11]. The anti-biofouling performance can be enhanced by ALGH crosslinking with Ca^2+^ ions [12].

ALGH is used as a dispersing agent for the colloidal processing of materials [13]. It is important to note that many advanced commercial dispersing agents cannot be used for biomedical applications due to their harmful effect on the human body. In contrast, ALGH is a biocompatible dispersant, which facilitates electrosteric particle dispersion. Moreover, ALGH was used as a capping agent for the synthesis and dispersion of inorganic nanoparticles [14,15]. ALGH exhibits remarkable adsorption properties, which are beneficial for surface modification of materials and dispersion of inorganic particles [16]. ALGH provides a versatile platform for the development of thin films for surface modification of materials. Anionic alginate was combined with various cationic polymers and other materials for the fabrication of films with advanced biomedical functionality using layer-by-layer (LBL) self-assembly [17,18,19]. This method involved deposition of alternating layers of negatively charged alginate and positively charged materials. Chemical modification of ALGH with catechol facilitated the fabrication of adherent films with antibacterial properties [20] by a spin coating method. Colloidal properties of ALGH were beneficial for the development of a dip coating method for surface modification of metals [21]. Electrophoretic deposition method (EPD) has been developed for the fabrication of ALGH films and coatings [22,23]. The EPD mechanism [22] is based on the dissociation of ALGNa into ALG^–^ and Na^+^:

ALGNa → ALG^–^ + Na^+^ (1)

The electric field provides the electrophoretic force moving ALG^−^ towards the anode, where the pH is low due to water decomposition and generation of H^+^ ions:

2H_2_O → O_2_ + 4H^+^ + 4e^−^ (2)

The negatively charged ALG^−^ is neutralized at the electrode by the positively charged H^+^ resulting in the ALGH film deposition:

ALG^–^ + H^+^ → ALGH (3)

The development of EPD of ALGH films provided a platform for the fabrication of composite films containing proteins, enzymes and other functional materials in the matrix of ALGH [23,24,25,26].

ALGH polymer has generated significant interest for the fabrication of composite materials, containing hydroxyapatite (HAP). It is important to note that natural bone is a composite organic-inorganic material [27]. HAP is an important material for biomedical applications, as its chemical composition is similar to that of the inorganic material part of natural bones. Synthetic HAP is a bioactive and biocompatible prosthetic material, bonding strongly to the bone and facilitating the formation of bone tissue on its surface. Many important investigations focused on influence of different factors on HAP stability [28,29] and fundamental aspects of biopolymer chemistry and physics for various applications [30]. Various polymers were analyzed [30,31,32] and it was found that ALGH is a very promising material for bioengineering due to its biocompatibility, biodegradability and other functional properties. Advanced functional properties of natural bone and other natural biomaterials result from unique design features, such as multilayer structure and hierarchical structural organization, containing various building blocks at different dimensional scales [33]. The use of organic-inorganic composites offers advantages of low temperature processing, which facilitates the fabrication of structures with hierarchical organization on the nano-, micro- and macro- scales and HAP crystallographic orientation similar to the natural bone [34,35]. The development of organic-inorganic nanocomposites eliminates multiple problems, related to the sintering of pure HAP implants, such as grain growth and HAP decomposition at elevated temperatures [36]. Moreover, various functional organic biomolecules and drugs can be incorporated into the organic-inorganic composites [37].

As noted above, ALGH belongs to the family of anionic polysaccharide polymers. It is known [38] that polysaccharides form interfaces between organic and inorganic components in bones and govern the crystallization of HAP nanoparticles. The anionic carboxylic groups of ALGH have an affinity for Ca^2+^ ions [39] and exhibit a strong influence on HAP nucleation and particle morphology. The size, shape and dispersity of HAP nanoparticles can be controlled using ALGH-mediated mineralization techniques [40,41,42]. Therefore, biomineralization and composite design are key aspects of ALGH applications for the development of bone substitute materials. ALGH-HAP composite particles were also used for the development of advanced vaccination techniques [43] and drug delivery [44].

This review is focused on the manufacturing and functional properties of ALGH-HAP organic-inorganic composites. The goal of this review is to emphasize innovative ideas in ALGH-HAP biocomposite technology, which are based on the unique properties of ALGH polymer and HAP. The current state of the research field of ALGH-HAP organic-inorganic composites is reviewed and key applications are outlined. Recent advances in materials synthesis, design, colloidal and electrochemical deposition and surface modification methods, fabrication of composites and clinical performance are described, which have enriched the science and technology of ALGH-HAP composites.

## 2. ALGH-HAP Films and Coatings

**Figure 1:**
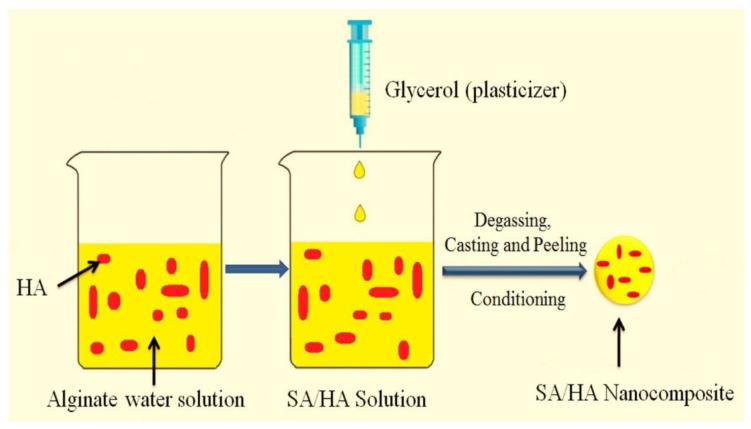
Casting of composite films from sodium alginate (SA) solutions, containing hydroxyapatite (HA) particles [45]. Used with permission. Copyright Elsevier 2018.

The literature data discussed above indicated that unique properties of ALGH polymer facilitated the development of ALGH films by different techniques, such as LBL, spin coating, dip coating and EPD. Such deposition techniques were further developed for the fabrication of ALGH-HAP films for advanced applications (Supplementary Materials, Table S1), The incorporation of HAP into the dip coated ALGH films resulted in improved coating stability and bioactivity [21]. The incorporation of 5 wt% HAP into ALGH films prepared by a casting method (Figure 1) resulted in enhanced mechanical and antibacterial properties and reduced water permeability [45].

**Figure 2:**
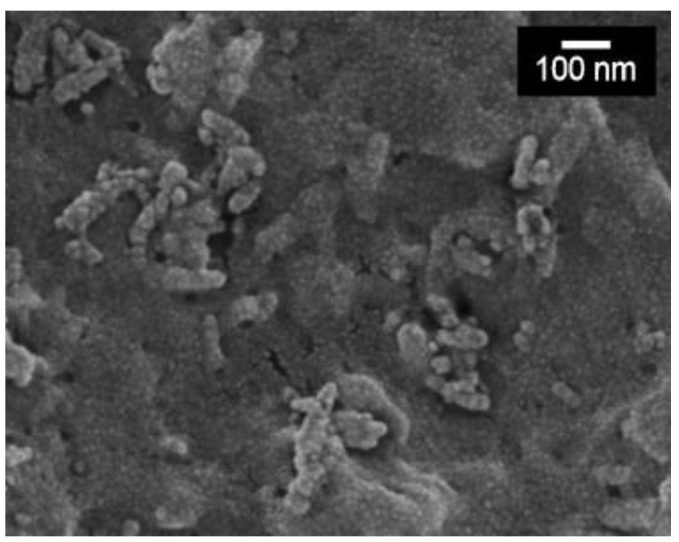
SEM image of the composite ALGH-HAP film obtained by EPD [22] from the 2 g/L sodium alginate solution containing 2 g L^−1^ HAP at a current density of 2 mA cm^−2^. Used with permission. Copyright Elsevier 2008.

EPD of ALGH provided a platform for the development of co-EPD of ALGH and HAP [22] from HAP nanoparticle suspensions in ALGNa solutions. It was found that ALG^−^ species adsorbed on HAP particles and facilitated their charging and dispersion. The deposition mechanism involved electrophoresis of HAP nanoparticles, containing adsorbed ALG^−^ toward the anode, where protonation of adsorbed ALG^−^ in reaction (3) resulted in particle coagulation and deposition on the electrode surface. The deposition of composite involved EPD of ALGH and HAP, containing adsorbed ALGH. The film forming and binding properties of ALGH facilitated the formation of adherent and continuous ALGH-HAP films (Figure 2) [22]. The film composition was varied by the variation of ALGH and HAP concentrations in the EPD bath [22]. Further development of this method resulted in EPD of bioactive composites containing ALGH, HAP and bioglass [46]. EPD of ALGH-HAP has been applied for the modification of 3-D porous Ti6Al4V scaffolds [47]. Co-EPD of ALGH and HAP facilitated better penetration of the coating material into the porous scaffold, compared to EPD of pure HAP. Coating thickness and depth were controlled by variation of applied voltage and deposition time and also depended on the suspending medium used [47]. The EPD of ALGH-HAP allowed the deposition of adherent and dense coatings at room temperature, avoiding the high temperature sintering process [47]. Coating microstructure was influenced by the HAP content. The increase in the HAP concentration in the EPD bath resulted in increasing content of HAP in coatings and the formation of agglomerates [48]. The negative charge of the carboxylic acid groups of ALGH was beneficial for the antibacterial properties of the composites [49]. EPD method was also used for the fabrication of ALGH-HAP composites, reinforced with carbon nanotubes [35]. The composite coatings were deposited as monolayers or multilayers and exhibited improved mechanical properties and corrosion protection of NiTi alloys in Ringer’s physiological solutions [35].

ALGH-HAP composite films were also deposited using an LBL technique on polycarbonate tubes, which were used as templates [50]. Obtained composites showed promising performance for soft tissue engineering applications. It was found that HAP imparted enhanced bioactivity to the composites [50]. ALGH was combined with cationic chitosan for the LBL fabrication of composite films, containing HAP [51]. The approach developed in this investigation was based on the ability to form stable HAP dispersions using alginate as an efficient dispersant. It was found that composites with high HAP loading improved the MC3T3 cell adhesion and proliferation [51]. Another strategy was based on the biomineralization of alginate-chitosan films formed using LBL self-assembly [52]. HAP was also used as a drug loading component for the alginate-polyvinyl alcohol films [53]. The composite films, containing amoxicillin as a drug showed high antibacterial activity against the Gram-positive and Gram-negative bacteria. Moreover, HAP facilitated regeneration of deteriorated bone segments due to periodontal defects [53].

## 3. ALGH-HAP Scaffolds

ALGH-HAP scaffolds have been developed from a variety of techniques [54,55,56,57,58,59,60] for biomedical applications (Supplementary Materials, Table S1). The scaffolds have been found to be extremely beneficial in tissue engineering [58,61,62,63] and drug delivery [64]. The scaffold properties are dependent on the concentrations of ALGH and HAP used, microstructure and the processing method. The porosity and density of the scaffolds are highly dependent on the ALGH concentration. The scaffold density increases as the alginate concentration increases [54]. The porosity decreases as the concentration of ALGH increases due to the increase of viscosity of ALGH at higher concentrations, which limits ALGH diffusion into the pores. Because ALGH diffuses into the pore network in the scaffold, it leads to pore closure as it covers the pores, leading to enhanced mechanical properties of the scaffold [54,55]. Crosslinking between the Ca^2+^ ions from HAP and the COO^−^ groups in ALGH further increases the mechanical properties [55] of the composite scaffolds. Such factors result in increasing mechanical properties of the scaffolds with increasing ALGH concentration. It was found that the ALGH coating is hydrophilic, leading to an increased swelling capacity and water absorption of the scaffold. Crosslinking ALGH with Ca^2+^ minimizes this hydrophilicity, decreasing the swelling capacity. High porosity increases the water absorption capacity of the scaffold. It was shown [54] that ALGH-HAP scaffolds enhance local bone healing because they do not damage liver or kidney functions nor induce carcinogenic or inflammatory effects. From the available literature, it becomes obvious that the development of materials processing techniques, control of scaffold composition, microstructure and HAP particle size plays an important role in the fabrication of advanced ALGH-HAP scaffolds [65,66,67] with enhanced biomedical performance.

**Figure 3:**
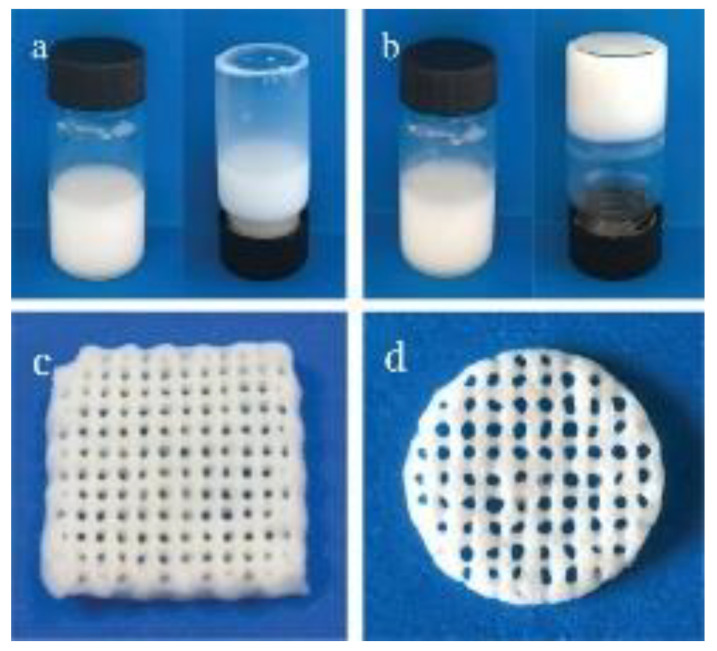
(**a**) The alginate-HAP suspension prior to crosslinking; (**b**) alginate-HAP suspension after crosslinking with D-Gluconic acid δ-lactone (GDL); (**c**,**d**) the ALGH-HAP scaffolds fabricated from the alginate-HAP suspension pre-crosslinked with GDL [61]. Used with permission. Copyright Wiley 2019.

Liu et al. fabricated ALGH-HAP scaffolds (Figure 3) by 3D printing [61]. It was found that the degree of crosslinking is a very important parameter to define the mechanical strength of the scaffold. With increasing crosslinking, the mechanical strength increases to a maximum until further crosslinking leads to an increase in the scaffold’s brittleness, which decreases the mechanical strength. A double crosslinking strategy has been developed which involved Ca^2+^ ions release from the HAP phase before printing and crosslinking in CaCl_2_ solution after printing. The use of HAP was beneficial for the optimization of porosity and improvement of mechanical strength. Mouse bone mesenchymal stem cells were shown to attach, grow and proliferate well on the ALGH-HAP scaffolds fabricated by 3D printing [61]. There is tremendous interest [68] in the development of scaffolds for jaw bone repair by 3-D printing.

Scaffolds have been developed for combined tissue engineering and local drug delivery. Several drugs have been combined into ALGH-HAP scaffolds, such as curcumin (Cur) [61] and chlorhexidine (CHX) [64]. Cur can be loaded onto SiO_2_ nanoparticles for incorporation into the scaffold. Cur is first released from the pores in the SiO_2_ nanoparticles, then released from the scaffold’s macropores. It was found that the greater the porosity of the scaffold, the more Cur released. As such, an increase of HAP content decreases the Cur release rates, as the increase in HAP decreases the pore size. Higher amounts of Cur loaded into the scaffolds lead to greater release rates, as the concentration difference between the scaffold and surrounding solution is greater [61]. CHX is bound to the scaffold through electrostatic interactions due to its positive charge and the negative charge of ALGH. As well, the swelling of the scaffold allows CHX to be absorbed into the scaffold. The more porous the scaffold, the more drug that can be absorbed. The bound water found in the scaffold helps prevent the drug release, as the water and CHX are bound with hydrogen bonding, leading to longer release times [64].

Investigations [69] revealed the benefits of ALGH-HAP scaffolds with fibrous microstructure, which mimics mineralized collagen fibrils in bone tissue. In this approach, the fibrous scaffolds were prepared by electrospinning and in situ synthesis of HAP. This material design systems offers benefits of reduced HAP agglomeration, improved HAP distribution in ALGH and enhanced biocompatibility [69]. Injectable fibrous ALGH-HAP scaffold materials were also prepared for the repair of bone defects [70].

**Figure 4:**
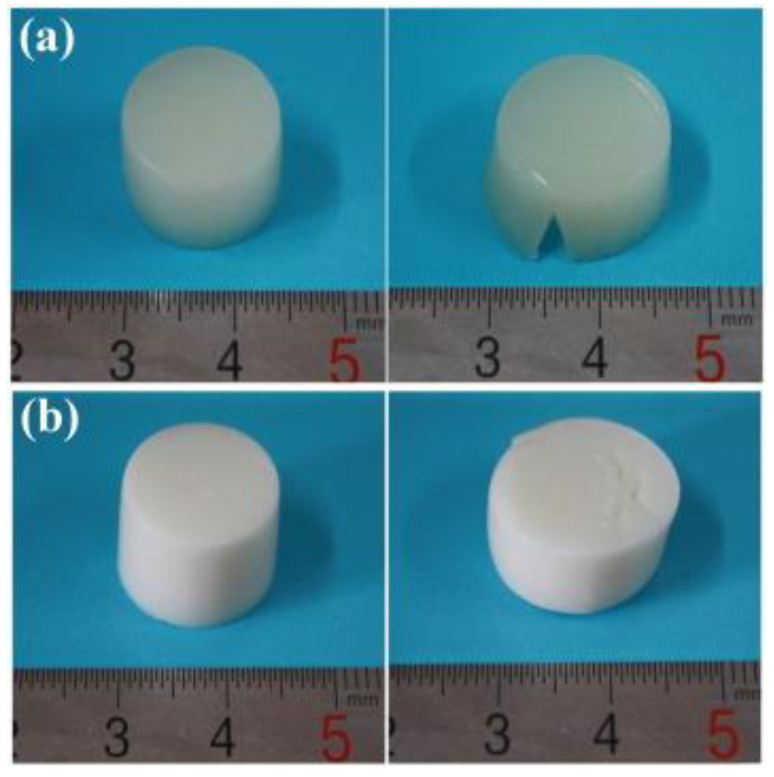
(**a**) Pure ALGH gel scaffold prior to (**left**) and following (**right**) compressive test; (**b**) ALGH-HAP scaffold containing 6% *w/v* HAP prior to (**left**) and following (**right**) compressive test [71]. Used with permission. Copyright Elsevier 2016.

ALGH-HAP scaffolds have been prepared in conjunction with several other polymers, such as gelatin (Gel) [71,72,73], chitosan (Chit) [72,74,75], fibrin [76], polyvinyl alcohol [77], polylactic acid [78,79], ethyl cellulose and poly(Ɛ-caprolactone) [80] and other polymers [81,82]. Figure 4 shows composite ALGH-HAP scaffolds, containing Gel.

The addition of Gel microspheres (mGel) to the scaffold further improved the mechanical properties of the scaffold. However, an excessive increase of mGel concentration decreases the mechanical properties due to its hydrophilic properties and the increased brittleness of the scaffold. The mGel were found to be homogeneously distributed throughout the scaffold walls. It slightly decreased the scaffold’s porosity by increasing the wall thickness. The incorporation of mGel increased the viscosity of the solution and, therefore, decreased the gelation time of the scaffold. The scaffold had a lower swelling capacity as the scaffold was more compact and less water could disperse into the scaffold [71]. The incorporation of HAP into the ALGH-Gel matrix facilitated the control of porosity and optimization of the pore size [83] for the penetration of cells. The incorporation of HAP into the ALGH-based scaffolds is beneficial not only for increasing scaffold stability but also for facilitating cell adhesion [78,84]. In the formation of ALGH-HAP-Chit scaffolds, ALGH’s COO^−^ groups can electrostatically interact with the NH_3_^+^ groups of Chit to weakly bind them together. As well, the NH_3_^+^ groups can interact with the PO_4_^3-^ groups of HAP, binding the scaffold together and forming a more compact scaffold. The decrease in porosity and degradation rate and increase in mechanical stability due to these increased interactions in the scaffold were reported [72]. The gelation rate of ALGH and the extent of crosslinking are key factors controlling rheological properties and the printability of the scaffolds throughout the 3D printing process [77]. Chit-ALGH polyelectrolyte complex porous structures were formed by freeze drying method and HAP coating was applied by a dip coating method [85]. ALGH coating on HAP-based scaffolds improved the biocompatibility, changed the roughness and microtopography of the scaffolds surface, which contributed to increase osteoblast adhesion and migration [86].

Advanced strategies have emerged as convenient methods for the fabrication of scaffolds with desired microstructures [79,87,88]. ALGH porous scaffold was used as a framework with uniformly distributed and interconnected pore structure and Chit-HAP composite solution was introduced into the pores of the ALGH framework to form polyelectrolyte complex scaffolds [87]. Highly porous ALGH-HAP microstructures with uniform HAP particle distribution were obtained using a freeze-drying technique [89,90]. ALGH-HAP composites with isotropic or anisotropic porosity were developed using two different freezing methods [91]. Osteoblast-like cells proliferated on both types of structures in a comparable manner [91].

Significant interest has been generated in the development of colloidal techniques, based on the use of ALGH as a dispersant for HAP [92]. Investigations highlighted benefits of 3-D printing of porous composite ALGH-HAP scaffolds, compared to sintered HAP scaffolds [93]. In situ mineralization of 3-D printed structures at mild conditions was beneficial for the porosity control [94].

Another promising strategy [95] is based on the mixing of calcium phosphate cement with ALGH and the in-situ transformation of the cement to the HAP phase for scaffold fabrication. This approach was used for drug delivery and bone tissue engineering [95]. The composites of HAP forming cements and ALGH, containing growth factors and other functional materials were developed for scaffold fabrication [96], which showed advanced bone-regeneration properties and mechanical stability. The mild processing conditions of the ALGH based composites, containing HAP forming cements, allowed the incorporation and controlled loading of proteins [97]. Moreover, injectable ALGH based scaffolds containing HAP forming cements were developed for the controlled release of antibiotics [98].

Among the interesting examples of scaffold applications is the development of ALGH based composite scaffolds, contained Sr-doped HAP. Such scaffolds showed enhanced compressive strength and promoted cell proliferation and osteoblast differentiation [99]. Alginate/Mg-doped HAP scaffolds were fabricated by “click” chemistry to mimic highly porous structures with the dimensional hierarchy of bone tissue [100]. The scaffolds allowed good preosteoblast cell attachment and proliferation [100]. 3D scaffolds containing Fe^2+^ doped nano-HAP-ALGH-Gel were prepared for magnetic resonance imaging based on non-invasive monitoring of bone tissue regeneration [101]. The incorporation of silver nanoparticles into ALGH-HAP scaffolds imparted them advanced antibacterial properties [102]. ALGH-HAP scaffolds prepared by 3-D printing provided a versatile platform for their loading with different drugs for controlled release [103]. ALGH-HAP scaffolds with controlled porosity were also prepared by a phase separation technique [104]. Such scaffolds showed a well-interconnected porous structure with an average pore size of 150 μm and over 82% porosity. The morphology of the scaffolds could be manipulated by tuning the quenching temperature during the preparation [104].

Cell-based tissue engineering is an important platform for the development of advanced scaffolds [105,106]. It is promising to create living functional tissues for bone regeneration [105]. ALGH-HAP scaffolds laden with stem cells were used for articular cartilage repair [107]. Testing results showed that such scaffolds can provide a high-quality biosubstitute for cartilage defects. Stem cells were incorporated in ALGH-HAP ink for the fabrication of scaffolds by a 3-D printing technology [108].

The use of dopamine modified ALGH has emerged [109] as an interesting strategy, particularly for the development of a gradient-structural scaffolds, which were designed to provide an optimized 3D environment for promoting cell growth. The use of dopamine modified ALGH enhanced its interactions with HAP and resulted in advanced mechanical properties of the scaffolds. The scaffolds showed optimized degradation rate to satisfy with the duration of new bone regeneration. In vitro and in vivo testing results [109] showed promising performance for implant applications.

## 4. ALGH-HAP Biocements

ALGH-HAP biocements have been developed as bone fillers for tissue engineering and orthopedics (Supplementary Materials, Table S1). Biocement setting involves reactions of mixed CaP phases with a liquid to form HAP. The use of ALGH in the biocement has a strong influence on the properties of the fabricated biocements [110,111]. ALGH-HAP biocements showed a strong organic–inorganic interface binding due to interlocking and crosslinking of the alginate strands [97]. It was found that ALGH becomes cross-linked by the calcium ions of the inorganic phase [110]. The setting time and microstructure of biocements are influenced by ALGH [111,112]. It was found that the ALGH additive increased the compressive strength, cohesion and toughness of the biocements [97,113,114,115]. Furthermore, the composite biocements exhibited favorable osteoconductivity and bioresorbability [113]. In another investigation, it was demonstrated that the addition of ALGH to biocements facilitated hardening reactions and improved the mechanical properties [116].

Investigations highlighted the importance of powder to ALGH liquid ratio (P/L) for obtaining injectability and hardenability [95]. The setting time, compressive strength, microstructure and porosity are dependent on P/L of biocements [115,116]. It was found that higher P/L ratios can result in lower setting times and higher compressive strength within a specific P/L range [116]. ALGH was added to promote the cohesion of the foamed biocement paste for implantation [117]. ALGH facilitated the incorporation of drugs and other functional biomaterials into the composite biocements [95,118,119]. The mild processing conditions allowed incorporation of proteins into the biocements with high loading efficiency [97]. Release studies in vitro showed that the protein release rate could be controlled [97]. Advanced biocements were also developed for cell delivery [110].

Various techniques have been developed for the fabrication of ALGH-HAP biocements [95,97]. Fibrous and injectable biocements have been found to be extremely beneficial in tissue engineering [95,97]. The addition of ALGH to biocements facilitated the development of advanced technologies and novel applications [118]. New printing strategies have emerged as convenient and rapid methods for various ALGH-HAP biocement applications [118]. Of particular interest is the ability of 3D bioprinting of soft cell-laden biocements [120,121] for bone tissue engineering.

ALGH-HAP biocements have been fabricated with the further addition of other materials, such as chitosan (Chit) [122,123], gellan gum [118], poly (lactic-co-glycolic acid) (PLGA) [124] and citric acid [125]. The addition of Chit increased the setting time of the cement and increased the compressive strength. As well, it made the cement more cohesive due to the rapid gelation of ALGH and Chit [122]. The addition of gellan gum facilitated the fabrication of printable materials [118]. PLGA additive resulted in enhanced compressive strength [124]. A synergistic effect of ALGH and citric acid [125] led to significantly shortened setting time and improved the anti-washout ability.

## 5. ALGH-HAP Gels

**Figure 5:**
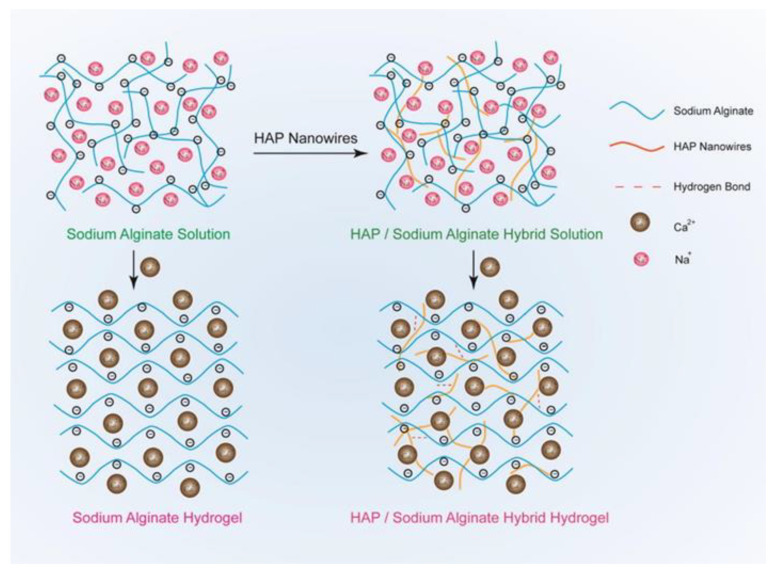
Schematic of the fabrication of ALGH gel (**left**) and the fabrication of ALGH-HAP nanowire gel (**right**) both crosslinked with Ca^2+^ [126]. Used with permission. Copyright Elsevier 2017.

ALGH-HAP gels have been developed for a wide variety of applications in tissue engineering and drug delivery (Supplementary Materials, Table S1). The amount of HAP in the gel has a strong influence on the gel’s mechanical properties. The gels can be crosslinked by adding Ca^2+^ ions from CaCl_2_ solutions, which bind to the carboxylate groups of ALGH through ionic interactions, forming an “egg-box” pattern [126,127], thereby further increasing the strength of the gel. HAP nanowires have been used to reinforce the gel (Figure 5), which resulted in significant improvement of the mechanical properties [126].

HAP nanowires interacted with the gel through electrostatic interactions from hydrogen bonding. These nanowires are highly flexible, as they have a very small diameter and are long, which is beneficial for improving the mechanical properties, as compared to brittle bulk HAP. These gels can easily be molded into a wide variety of shapes and after the removal of the mold, the gel holds its shape well. The gels are highly elastic and have an increased tensile and compressive strength compared to pure ALGH gels. The ALGH-HAP gels have a much improved continuous porous structure compared to ALGH gels and the composite gel demonstrates a much higher density, similar to that of natural bone [126]. The composite gel porosity was influenced by the inorganic phase content [126].

ALGH-HAP gels have been developed by the mineralization of HAP during the gelation process using brushite seed crystals. Ca^2+^ ions diffuse towards the bulk of the solution causing gelation as they diffuse. Addition of PO_4_^3–^ ions decreases the gelling velocity due to the consumption of Ca^2+^ ions during gelation and the formation of HAP [128]. This investigation revealed crystal growth resulting from Ostwald ripening [128].

**Figure 6:**
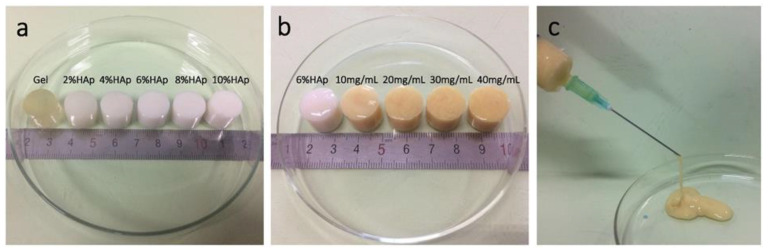
(**a**) ALGH-HAP gel with increasing amounts of HAP; (**b**) ALGH-HAP-CM gels containing 6% HAP with increasing amounts of CM; (**c**) the injection of ALGH-HAP-CM gel (6% HAP and 5% CM) from a syringe needle [130]. Used with permission. Copyright Elsevier 2018.

ALGH-HAP gels showed interesting stimuli-dependent properties that make them useful for drug delivery applications [129]. Applying a voltage to the ALGH-HAP gel causes a size change. It was shown that the size of the gel decreases with increasing voltage. In the proposed mechanism, H_3_O^+^ ions are generated and their movement decreases the size of the gel due to electro-osmosis and electrophoresis [129]. Gel swelling is highly pH-dependent, with lower HAP content showing greater swelling. The swelling is dependent on the hydrophilic groups, crosslinking density and the HAP distribution throughout the gel. With a high HAP concentration, there are fewer hydrophilic groups available as they are bonded to the HAP and there is a high distribution of HAP throughout the gel, which all work together to develop low swelling of the gel. In acidic media (pH = 4), the carboxylate groups of ALGH become protonated, so there is lower hydrophilicity, leading to a low amount of swelling. However, in basic media (pH = 7.4), the carboxylate groups repel each other, giving greater hydrophilicity and leading to an increased amount of swelling. As well, the degree of crosslinking density decreases in basic media, which further increases the degree of swelling. The greater degree of swelling is beneficial for cell attachment and proliferation, as there is more space in the gel network for the cells to grow and proliferate [129]. Figure 6 shows ALGH-HAP gels with and without calcium carbonate microshere (CM) additives, which improve mechanical properties of the gels.

A variety of drugs, such as tetracycline [127,130,131], dimethyloxalylglycine [127], silver sulfadiazine [131] and doxorubicin (DOX) [132], can be encapsulated into ALGH-HAP gels for their release. Hydrogen bonding allows the drugs to bind to the gel network. The release rates are increased when the gel has a greater degree of swelling and increased swelling rate. This increased swelling is found in gels with low amounts of HAP, as these gels have greater pore spaces and larger pores. It was found that the drug release rate is proportional to the swelling of the gel [127]. Despite the increase of swelling of the gel in basic media, DOX shows a decreased release rate as the drug begins to interact with ALGH’s carboxylate groups, which restricts its release from the gel. As such, DOX shows the greatest release rate at a pH = 5. At this pH, there is an increased dissolution of HAP, which facilitates the diffusion of DOX from the gel [132]. The drugs show an initial burst release, then maintain a sustained release. The amount of drug release depends on the solubility of the drug, as more soluble drugs release a greater amount from the gel [131].

ALGH-HAP gels have been developed incorporating other compounds such as collagen (Col) [133], gelatin (Gel) [134] and chitosan (Chit) [135]. The novel Col-ALGH-HAP gel was developed by forming an injectable Col-ALGH gel followed by nucleating HAP onto the Col fibers, leading to adequate mechanical strength to act as a bone substitute. The Col fibers serve to reinforce the gel matrix giving the increased strength [133]. The Gel-ALGH-HAP gel demonstrated a highly adhesive gel matrix allowing it to fix to the surface of bone. It can be loaded with vancomycin hydrochloride to minimize the inflammatory response and demonstrate an antibacterial response [134]. Chit helps to stabilize the gel matrix through the formation of a polyelectrolyte complex due to the positive charge found on Chit’s amino groups and the negatively charged ALGH from the carboxyl groups [135].

## 6. ALGH-HAP Beads

**Figure 7:**
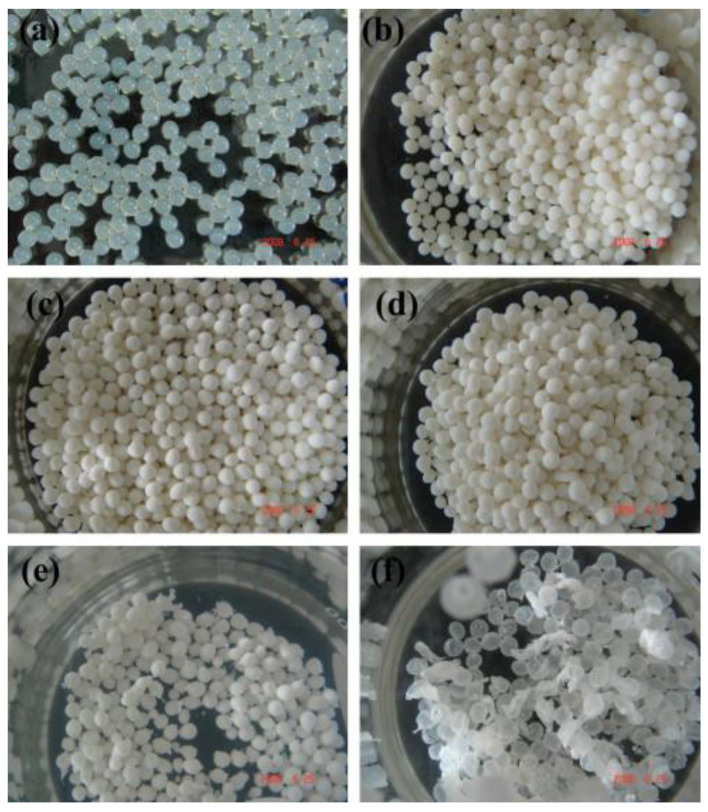
(**a**) Pure ALGH beads; (**b**–**f**) ALGH-HAP beads with increasing HAP content [138]. Used with permission. Copyright Elsevier 2010.

ALGH-HAP beads have been developed (Supplementary Materials, Table S1) for tissue regeneration and drug delivery of a wide variety of compounds. The beads are typically spherical in shape (Figure 7), in the range of 2.5–4 mm in diameter (Figure 7) [136,137,138].

**Figure 8:**
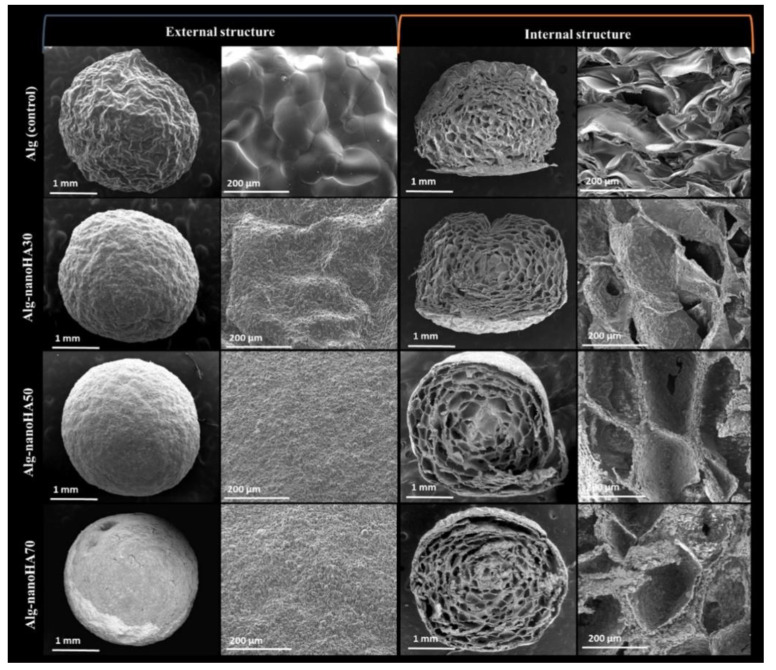
SEM images of ALGH-HAP beads with increasing HAP content (top to bottom), showing the external structure (**left**) and internal structure (**right**) at different magnifications. Note the increasing roughness as the HAP content increases [136]. Used with permission. Copyright Elsevier 2019.

The composite beads demonstrated a slightly roughened surface and nanoHAP was distributed homogeneously throughout the bead, which gives the bead a white color. Irregular, interconnected pores were found throughout the bead. These pores allowed the beads to retain large amounts of water, which allows the diffusion of nutrients throughout the bead for cell growth. The addition of nanoHAP is able to promote water diffusion into the bead due to the carboxyl and hydroxyl groups of ALGH, which can interact with the water molecules. The addition of nanoHAP increased the degree of crosslinking in the network by increasing the ionic strength. Due to this increase in crosslinking, the amount of swelling of the bead decreased with increasing nanoHAP content. This decrease in swelling further serves to restrict water and nutrient flow through the bead. The ALGH-HAP composite beads swell less in acidic media than basic media due to the protonation of the ALGH carboxylate groups in acidic media and the repulsion of the carboxylate groups in basic media. When the beads are placed in simulated body fluid, apatite mineral growth is seen on the surface of the beads. NanoHAP contains hydroxyl groups which serve as nucleation sites on the bead for this deposition [136]. Figure 8 shows the microstructure of ALGH-HAP beads with different HAP content.

ALGH-HAP beads have been produced with the addition of a phenolic additive which reduced the degradation behavior of the beads in the presence of lysozyme, compared to ALGH beads and the addition of HAP further significantly reduced the degradation rate. They both also increase the mechanical properties of the beads. This modification strategy also serves to decrease the swelling ratio of the bead. These properties of the modified beads are due to the additional crosslinking providing more structural support, which serves to decrease the degradation rate and swelling. Chemical interactions between the functional^−^ groups of the organic components and HAP further increase the degree of crosslinking in the bead and improve these properties [137].

Multiple different drugs have been encapsulated in ALGH-HAP beads for drug delivery, such as diclofenac sodium (DS) [138], ibuprofen(Ibu) [139] and anaesthesinum [140]. The drugs are released from the beads through a diffusion process [138,139]. The negatively charged carboxylate group found on DS can bind to the Ca^2+^ crosslinking the matrix, which binds DS to the beads. Increasing ALGH concentrations allows for improved loading of DS in the beads. The beads have a cabbage-like pore structure, which forms barriers preventing drug release from the beads. To minimize drug burst release, the dissolution rate could be decreased and bead disintegration could be avoided. This can be achieved by increasing the HAP content, which minimizes the initial swelling and slows the crosslink breakdown [138].

Ibu can be loaded into ALGH-HAP beads and Ibu binds to the beads through hydrogen bonding between the carboxyl groups of Ibu and the carboxyl groups of ALGH. The carboxyl groups of both Ibu and ALGH are deprotonated, which greatly increases the solubility of Ibu in the media, as well electrostatic repulsion between these deprotonated carboxyl groups leads to a fast release of the drug. However, the addition of HAP controls this release behavior in the same manner as DS. In acidic media, the low swelling restricts the release of the beads, as well the carboxyl groups of both Ibu and ALGH remain protonated; therefore, the hydrogen bonding further slows their release from the beads [139].

## 7. Conclusions and Future Trends

The literature data described in this review indicated that ALGH based materials loaded with HAP are promising for bone tissue regeneration, treatment of bone defects, surface modification of implants, drug delivery, endodontics and other biomedical applications. The synergy of functional properties of ALGH and HAP offers a strong potential for the development of bone substitute materials with advanced microstructure and properties. The bioactivity of ALGH and HAP composites provides a platform for advanced implant applications. Composite materials have been developed in different forms, such as films, coatings, scaffolds, gels, beads and biocements. ALGH-HAP composites can be loaded with other functional materials for the fabrication of new composites with advanced functionality.

New coating deposition mechanisms based on pH dependent charge and solubility of ALGH as well as ALGH adsorption on HAP are key factors for new emerging developments in the field of surface modification of biomedical implants. ALGH can be used as a biocompatible capping, dispersing and film forming agent for different film deposition techniques. Further advances can be achieved by the development of film crosslinking techniques to improve the chemical stability of the coatings. EPD offers the potential for co-deposition of ALGH and HAP with other functional biomaterials. Progress in this area can also be achieved by the optimization of ALGH molecular size, chemical modifications of ALGH molecules, optimization of the size and shape of HAP particles and their dispersion.

Many examples throughout literature display the beneficial use of ALGH-HAP scaffolds for tissue engineering and drug delivery applications, combining the properties of the organic and inorganic components for superior scaffold properties. Different crosslinking methods are being researched to improve the mechanical properties and swelling of the scaffold. Much current research is focused on the 3D printing of such scaffolds, the optimization of bioink compositions, as well as the preparation of ALGH-HAP scaffolds combined with other polymers to augment the scaffold properties. New technologies for 3D printing of ALGH-HAP scaffolds facilitate the control of porosity, which is one of the key factors for successful biomedical applications. As such, many further advances can be made in these areas. Cell-based tissue engineering is a promising platform for the development of advanced scaffolds. It is promising to create living functional tissues for bone regeneration.

ALGH-HAP biocements demonstrate excellent properties for bone filling applications in tissue engineering, with the possibility for local drug delivery in vivo. They display suitable cell growth and proliferation and setting times for clinical applications. The addition of other compounds to the ALGH-HAP cement can further modify the cement properties. Future research can develop cements with stronger mechanical properties for the potential use in load-bearing applications, as the mechanical strength of current cements is only suitable for non-load-bearing applications. Further advances can be achieved by the development of fully biodegradable cements to improve their use in tissue engineering applications.

ALGH-HAP gels have many potential applications due to their moldability into a wide variety of shapes, their elasticity and ease of gelation in solution. The swelling of the gel is easily controlled by the degree of crosslinking of the gel and the pH of the media it is placed in. Much research is focused on the controlled release of drugs from these gels for local drug delivery. Further advances can be achieved through the optimization of the morphology of HAP dispersed in the gel.

Significant interest has been generated for the use of ALGH-HAP beads in controlled delivery of different drugs. The porous structure that develops and low swelling is ideal for sustained drug release, minimizing the initial burst release. However, more research can further optimize the drug release properties for more controlled release.

## Data Availability

Not applicable.

## Supplementary Materials

The following is available online at https://www.mdpi.com/article/10.3390/polym13183070/s1, Table S1. Applications of ALGH-HAP composites.
