# Supplementary material for: Alginic Acid Polymer-Hydroxyapatite Composites for Bone Tissue Engineering
Source: Polymers (Basel). 2021 Sep 11;13(18):3070. doi: 10.3390/polym13183070 (PMC8471633; doi:10.3390/polym13183070)
Supplement: Supplementary file 1 [file polymers-13-03070-s001.zip › polymers-1369410-SI.pdf]

polymers

Supplementary Information

Alginic acid polymer-hydroxyapatite composites for bone tissue engineering

Rebecca Sikkema, Blanca Keohan and Igor Zhitomirsky*

Department of Materials Science and Engineering, McMaster University, Hamilton, ON L8S4L7, Canada; sikkemar@mcmaster.ca (R.S.); keohanb@mcmaster.ca (B.K.)
*Correspondence: zhitom@mcmaster.ca (I.Z.)

Citation: Sikkema , R.; Keohan , B.; Zhitomirsky, I. Alginic acid polymer-hydroxyapatite composites for bone tissue engineering . *Polymers* **2021**, *13*, 3070. https://doi.org/10.3390/polym13183070

Academic Editor: Firstname Lastname

Publisher's Note: MDPI stays neutral with regard to jurisdictional claims in published maps and institutional affiliations.

Copyright: © 2021 by the authors. Licensee MDPI, Basel, Switzerland. This article is an open access article distributed under the terms and conditions of the Creative Commons Attribution (CC BY) license (http://creativecommons.org/licenses/by/4.0/).

Table S1. Applications of ALGH-HAP composites

Composite	Performance	Reference
Coating	Improved coating stability and bioactivity	[21]
Coating	Improved mechanical properties and corrosion protection	[35]
Film	Enhanced mechanical and antibacterial properties, reduced water permeability	[45]
Film	Enhanced bioactivity	[50]
Film	Improved MC3T3 cell adhesion and proliferation	[51]
Film	Improved drug delivery and bone defect healing properties	[53]
Scaffold	Enhanced mechanical properties and improved bone healing	[54]
Scaffold	Enhanced mechanical properties and biocompatibility	[55]
Scaffold	Improved porosity and biocompatibility	[61]
Scaffold	Enhanced biocompatibility	[65]
Scaffold	Enhanced biocompatibility and improved microstructure	[66]
Scaffold	Enhanced biocompatibility	[67]
Scaffold	Enhanced bone repair	[68]

Scaffold	Reduced HAP agglomeration, improved microstructure and biocompatibility	[69]
Scaffold	Improved injectability for repair of bone defects	[70]
Scaffold	Reduced swelling	[71]
Scaffold	Reduced degradation rate	[72]
Scaffold	Enhanced cell adhesion	[78]
Scaffold	Optimized porosity and improved biocompatibility	[83]
Scaffold	Enhanced cell adhesion	[84]
Scaffold	Improved biocompatibility and cell adhesion	[86]
Scaffold	Improved and optimized microstructure	[88]
Scaffold	Enhanced cells proliferation	[91]
Scaffold	Enhanced porosity control, improved biocompatibility	[94]
Scaffold	Advanced drug delivery properties	[95]
Scaffold	Enhanced bone regeneration properties	[96]
Scaffold	Controlled loading of proteins	[97]
Scaffold	Controlled release of antibiotics	[98]
Scaffold	Enhanced compressive strength and cell proliferation	[99]
Scaffold	Improved microstructure and cell proliferation	[100]

Scaffold	Enhanced bone tissue regeneration	[101]
Scaffold	Enhanced antibacterial properties	[102]
Scaffold	Controlled drug release	[103]
Scaffold	Improved morphology control	[104]
Scaffold	Improved bone regeneration properties	[105]
Scaffold	Improved articular cartilage repair	[107]
Scaffold	Optimized microstructure promoting cell growth	[109]
Biocement	Enhanced mechanical properties and biocompatibility	[110]
Biocement	Enhanced mechanical properties and biocompatibility	[111]
Biocement	Enhanced osteoconductivity and bioresorbability	[113]
Biocement	Enhanced mechanical properties	[114]
Biocement	Enhanced mechanical properties and cohesion	[115]
Biocement	Enhanced mechanical properties and cohesion	[116]
Biocement	Drug delivery properties	[118]

Biocement	Drug delivery properties	[119]
Biocement	Increased compressive strength and cohesion	[122]
Biocement	Shortened setting time and improved the anti-washout ability	[125]
Gel	Enhanced mechanical properties	[126]
Gel	Enhanced biocompatibility and mechanical properties	[127]
Gel	Drug delivery properties	[129]
Gel	Drug delivery properties	[130]
Gel	Drug delivery properties	[131]
Gel	Enhanced mechanical properties	[133]
Gel	Antibacterial properties	[134]
Beads	Improved biocompatibility	[136]
Beads	Improved biocompatibility	[137]
Beads	Drug delivery properties	[138]
Beads	Drug delivery properties	[139]
Beads	Drug delivery properties	[140]
